# Classification of Valleytronics in Thermoelectricity

**DOI:** 10.1038/srep22724

**Published:** 2016-03-14

**Authors:** Payam Norouzzadeh, Daryoosh Vashaee

**Affiliations:** 1 Electrical and Computer Engineering Department, Montheith Research Center, North Carolina State University, Raleigh, NC 27606, USA

## Abstract

The theory of valleytronics as a material design tool for engineering both thermal and electrical transport properties is presented. It is shown that the interplay among the valleytronics parameters such as the degeneracy of the band, intervalley transitions, effective mass, scattering exponent, and the Fermi energy may deteriorate or ameliorate any or all of the main thermoelectric properties. A flowchart classifying the different paths through which the valleytronics can influence the thermoelectric figure-of-merit ZT is derived and discussed in detail. To exemplify the application of the flowchart, valleytronics in four different semiconductors, Mg_2_Si, Si_0.8_Ge_0.2_, Al_x_Ga_1−x_As and clathrate Si_46_-VIII were studied, which showed different trends. Therefore, a degenerate multivalley bandstructure, which is typically anticipated for a good thermoelectric material, cannot be a general design rule for ZT enhancement and a detailed transport study is required to engineer the optimum bandstructure.

The approach to control over the valley degree of freedom is known as valleytronics. Valleytronics and the ability to control the transport of charge carriers via multiple valleys has found many applications in semiconductors^1^,^2^,^3^,^4^. Valleytronics can be also considered as a material design tool through which a multivalley bandstructure is engineered for improving the thermoelectric power factor. The efficiency of a thermoelectric convertor is a function of the dimensionless thermoelectric figure-of-merit ZT which is quantified as ZT = S^2^σT/κ, in which S, σ, T, and κ are the Seebeck coefficient, electrical conductivity, temperature and total thermal conductivity, respectively. S^2^σ is considered as the thermoelectric power factor. The principal challenge to enhance the thermoelectric performance is decoupling of S, σ, and κ, which are strongly interrelated. Different approaches have been adopted to improve thermoelectric properties such as adjusting the doping concentration^5^,^6^,^7^, modification of carrier mobility^8^,^9^,^10^, variation of band structure^11^,^12^,^13^,^14^, increasing the asymmetry of the differential electrical conductivity by energy filtering^1^,^15^,^16^,^17^, reduction of lattice thermal conductivity by nanostructuring^18^,^19^,^20^,^21^, introducing point defect to reduce the thermal conductivity^5^,^22^, convergence of multivalley bands to enhance the Seebeck coefficient^11^,^12^,^23^,^24^, and different bulk nanostructuring techniques to reduce the thermal conductivity and in some cases improve the power factor simultaneously^25^,^26^,^27^,^28^.

While most advances in thermoelectric materials research have been through the reduction of the thermal conductivity without or with smaller deterioration of the thermoelectric power factor, for many power generation applications, such as waste heat recovery in automobiles, it is more important to improve the thermoelectric power factor than reducing the thermal conductivity and valleytronics can provide a road-map to achieve this need. It is commonly accepted that the power factor enhances as the number of valleys near the energy band edges increases. Consequently, there have been increasing efforts to create bandstructures with multiple valleys or with high degeneracy aiming to enhance the thermoelectric power factor. Similarly, temperature assisted band convergence of several degenerate valleys within the bandstructure of the semiconductor which possesses a multivalley electronic bandstructure can improve the thermoelectric power factor^29^,^30^.

Formation of the multivalley bandstructure as a valleytronics technique has been already employed to improve the thermoelectric performance of several materials such as, PbTe_1−x_Se_x_^23^, Zr_3_Ni_3−x_Co_4_Sb_4_^31^, and Mg_2_Si_1−x_Sn_x_^32^ compounds. Nevertheless, it should be noted that the multivalley bandstructure, as we will discuss, does not always improve ZT. For example, intervalley scattering resulted from the multivalley band structure can lower the carrier mobility and reduces the beneficial effect of multivalley contribution in carrier transport. As we will discuss, the thermal conductivity can be also influenced by the degeneracy of the band.

The aforementioned effects raised some questions and motivated us to address them. For example, under what conditions the power factor enhances? Is there any effect on the electronic or lattice thermal conductivity due to the intervalley scattering? Are these effects beneficial or detrimental for thermoelectric performance of the materials? We will discuss these questions in a conceptual manner and then use a multi-band Boltzmann transport theory to demonstrate different scenarios of the detrimental and beneficial effects of the multivalley bandstructure in four different materials including Mg_2_Si, Al_x_Ga_1−x_As, Si_0.8_Ge_0.2_, and type-VIII clathrate Si_46_. Section II describes the theoretical background. The results are discussed in Section III. The comparison of Mg_2_Si, Al_x_Ga_1−x_As, Si_0.8_Ge_0.2_, and type-VIII clathrate Si_46_ are discussed in Section IV. The summary and conclusion is presented in Section V.

## Theoretical background

The multivalley band structure affects directly and indirectly the interdependent thermoelectric variables S, σ, κ_e_, and κ_l_. It can directly affect the Seebeck coefficient through the density of states effective mass *m*_*dos*_ and intervalley scattering, carrier mobility through intervalley scattering, and electronic thermal conductivity through intervalley scattering. It can also affect indirectly both the lattice part of thermal conductivity and the carrier mobility through 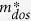. In order to show such interdependencies, we may write the following relations for a single valley parabolic band:


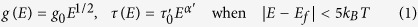


where *E*, *g*(*E*), *τ*(*E*), and *α*′are the energy, density of states, energy dependent scattering time and the scattering exponent for a single valley band, respectively. 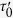 is an energy independent constant that depends on the scattering mechanism and the material properties. It should be noted that *τ*(*E*) can have a more complex dependency to energy; however, since it does not typically change rapidly with the energy change of a few k_B_T, one may approximate it by a power law function around the Fermi energy E_F_ as shown in (1).

For a multivalley bandstructure, assuming intervalley relaxation time of 
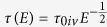
, in which *τ*_0*iv*_ is the energy independent constant for the intervalley scattering, we may modify relations (1) according to:


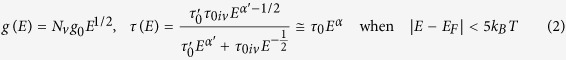


in which *N*_*v*_ is the number of equivalent valleys in the band. Here, *α* is the effective scattering exponent that describes the energy dependency of the total relaxation time. The main thermoelectric quantities can be calculated from the following formulas^11^:


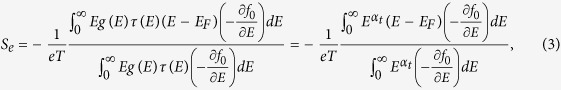



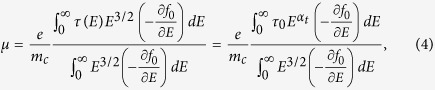



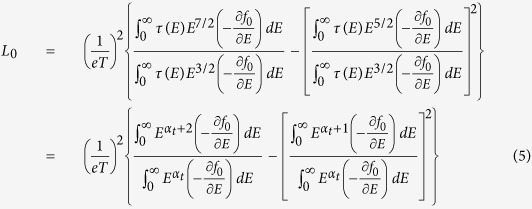



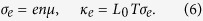


In the aforementioned formulas, *μ* is the electron mobility, *S*_*e*_ is the electron Seebeck coefficient, *L*_0_ is the Lorentz number, *σ*_*e*_ is the electrical conductivity, *κ*_*e*_ is the electronic part of thermal conductivity, *m*_*c*_ is the conductivity effective mass, *n* is the carrier concentration, and *E*_*F*_ is the Fermi energy f_0_ represents the Fermi-Dirac distribution at equilibrium. Here *α*_*t*_ = *α* + 3/2, where *α* is the scattering exponent as discussed before.

The above equations are assuming parabolic band approximation. If the band is not parabolic, the density of state has different energy dependency than *E*^1/2^; therefore, α_t_ ≠ α + 3/2 and one may find an effective energy exponent by expanding the density of states versus energy near the Fermi surface. For example, in the case of bandstructures with small non-parabolicity, one can use a single non-parabolicity parameter as *β* to explain the band strcuture according to the Kane’s model^33^ such that 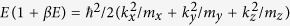, where *m*_*x*_, *m*_*y*_, and *m*_*z*_ are the diagonal elements of the effective mass tensor. In this case, *Eg*(*E*)τ(*E*) can be approximated by 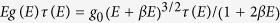
11. Expanding versus *E* around *E* = 0, we can express 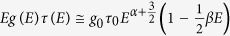. The term 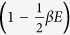 is the correction due to the non-parabolicity of the band.

In this section, for a clear visualization, we will focus on the valleytronics of the parabolic band and its effects on thermoelectric properties. However, we will solve numerically the transport equations considering the non-parabolic band approximation through the Kane’s model for the selected materials.

Thermal conductivity was calculated using the Steigmeier approach^34^. Using the Debye model the contribution of the optical modes to the thermal conductivity was excluded^35^. The model assumes linear spectrum for the acoustic phonons and constant energy for the optical phonons. Phonon-phonon scattering, phonon-electron scattering, and point defect scattering mechanisms with separate relaxation times were accounted for in the calculation of the thermal conductivity. The formalism introduced by Callaway is used to calculate the lattice thermal conductivity κ_*l*_^36^,^37^:


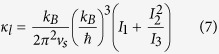


in which:


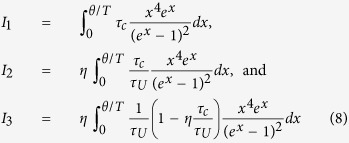



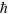
 and k_B_ are Planck’s and Boltzmann constants, respectively. The phonon angular frequency, the phonon group velocity (sound velocity), Debye temperature and absolute temperature are indicated by *ω*, *v*, *θ*, and *T*, respectively. *η* is the ratio of the Umklapp three-phonon relaxation time (*τ*_*U*_) to normal three phonon relaxation time (*τ*_*N*_). *τ*_*C*_ is the total relaxation times calculated using the Matthiessen’s rule.

**Figure 1:**
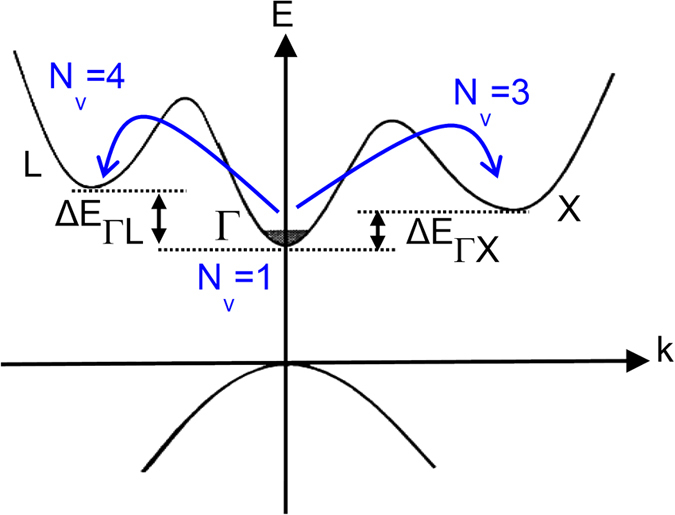
Non-equivalent intervalley scattering in AlGaAs material system. X and L points are degenerate; hence, both equivalent and non-equivalent intervalley scattering of electrons at each valley and between two valleys can occur, respectively. The depicted intervalley scatterings are from Γ to L and X valleys via absorption of an optical phonon.

In order to evaluate the transport properties, the relaxation time for each scattering mechanism must be determined. Assuming the scattering mechanisms are independent, the total relaxation time can be estimated from Matthiessen’s rule. We have employed two sets of scattering mechanisms. The first set includes the scattering mechanisms that affect the electrical properties such as charge mobility, electronic thermal conductivity and Seebeck coefficient. In this step, we calculated the ionized impurity, electron-phonon (acoustic and optical), and intervalley scatterings^33^. The second set includes the scattering mechanisms that influences the thermal conductivity such as 3-phonon, point defect (PD), and electron-phonon scatterings^34^. The multivalley band structure indirectly affects the scattering mechanisms by changing the energy of carriers and the density of states effective mass. The relaxation times for different scattering mechanisms can be found in the literature^11^,^33^. However, due to the importance of the phonon scattering by electrons and electron intervalley scatterings by phonons, we express their relations. We applied the following expression for the phonon-electron relaxation time^38^,^39^:


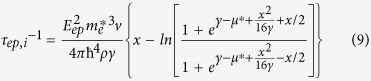


where 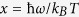, 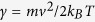, *ρ* is the mass density, μ* is the reduced Fermi energy, i.e. *E*_*F*_/*k*_*B*_*T*, E_ep_ is the electron-phonon deformation potential, and *v* is the sound velocity. At presence of multivalley band structures, the intervalley scattering mechanisms for charge carriers should be also taken into account. The acoustic and optical phonons can scatter charge carriers from one valley to another one. In case of degenerate band structure, the charge carriers can scatter from one degenerate valley to another as well. Equivalent and non-equivalent intervalley scatterings can be important for both direct and indirect semiconductors. A schematic representation of the intervalley scattering is illustrated in the Fig. 1.

Evidently, intravalley and equivalent intervalley scatterings are stronger than non-equivalent intervalley scattering as the formers occur by acoustic phonons and the latter requires optical phonons. The scattering rates for equivalent and non-equivalent valleys for electron transfer from *i* to *j* valley are given, respectively, by the following relations^33^:


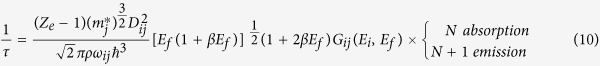



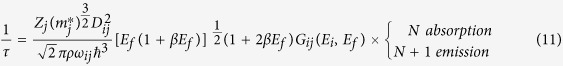


in which E_f_ is the final energy, i.e. 
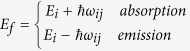
and E_i_ is the initial energy (prior to the scattering). Note that here i refers to the initial state and not the valley index as in ij subscript. *β* is the non-parabolicity parameter. *G*_*ij*_ is defined as 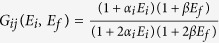. *Z*_*e*_, *D*_*ij*_, *ω*_*ij*_, *ρ* are the number of equivalent valleys, acoustic phonon deformation potential, the phonon frequency which allows the intervalley scattering, and mass density, respectively. In case of non-equivalent intervalley scattering, *Z*_*e*_ − 1 is replaced by *Z*_*j*_ which is the number of available final valleys for scattering.

It is notable that for charge carrier transport properties two types of effective masses are discussed. First is the density-of-states effective mass *m*_*dos*_ which affects the Seebeck coefficient and the second is the conductivity effective mass *m*_*c*_ which influences the carrier mobility and electrical conductivity. These effective masses are defined by the following relations:


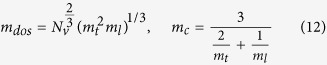


where *m*_*t*_ and *m*_*l*_ are the transverse and longitudinal principal band edge effective masses. The conductivity effective mass relation is valid provided that the material has an isotropic conductivity as in the case of cubic materials. In practice, creating large density-of-states effective mass through either band structure engineering^11^ or nanostructuring is considered as a method to enhance the Seebeck coefficient. However, a large effective mass results in simultaneous decrease of carrier mobility^40^. In summary, the Seebeck coefficient of a material with parabolic bandstructure (or near parabolic bandstructure) is enhanced by (1) large effective mass tensor elements (*m*_*t*_, *m*_*l*_,), which reduces the mobility, (2) large number of equivalent valleys in the band, which increases the density of states effective mass and reduces the mobility due to intervalley scattering, and (3) large positive exponent of the relaxation time *τ*, which may decrease or increase the carrier mobility depending on other scattering parameters.

All different kinds of electronic scattering rates scale with the density of states^33^. Since the density of states is directly proportional to the degeneracy of the valley *N*_*v*_, the scattering rates in a multivalley semiconductor scale with *N*_*v*_, which means that the relaxation times are scaled with energy as *τ*(*E*) = *τ*_0_*E*^*α*^/*N*_*v*_ following eqs 1 and 2. α is the scattering exponent that varies with the type of scattering mechanism. For example, this exponent equals to 3/2 and −1/2 for ionized impurity and acoustic phonons scatterings, respectively. Table 1 lists the exponent of various scattering mechanisms. As we will discuss, for the case of simple parabolic band for a bulk material, all types of scattering mechanisms, except the ionized impurity scattering, lead to the reduction of Seebeck coefficient.

**Figure 2:**
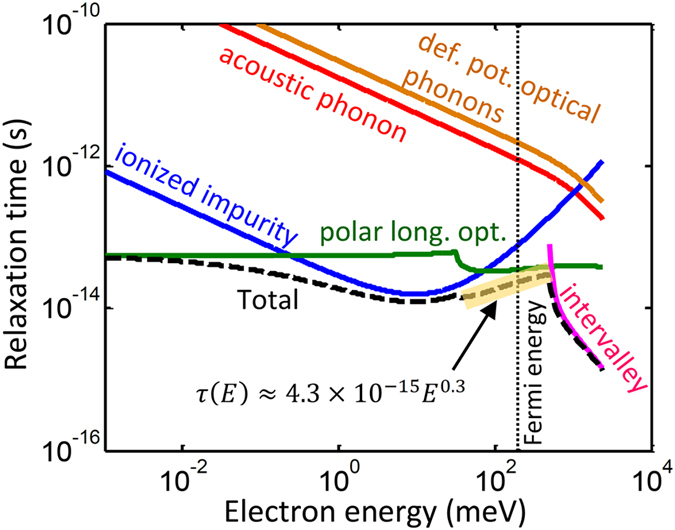
Relaxation times versus energy for different electron scattering mechanisms in Al_0.12_Ga_0.88_As for doping concentration of 1.44 × 10^19^ cm^−3^ at 1000 °C.

As an example, Fig. 2 shows the electron relaxation time versus energy for several scattering mechanisms in Al_0.12_Ga_0.88_As. These include scatterings due to acoustic phonon, ionized impurities, polar longitudinal optical phonons, deformation potential of optical phonons, and intervalley scattering mechanisms. The calculations were prformed for doping concentration of 1.44 × 10^19^ cm^−3^ at 1000 °C. The black dashed line represents the total relaxation time calculated using Matthiessen’s rule. The corresponding Fermi energy is 196 meV, which is shown by a black dotted vertical line. Since the transport happens near the Fermi energy, the highlighted segment of the total relaxation time was used for fitting the relaxation time to the power law function *τ*(*E*) = *τ*_0_*E*^*α*^. In this case, *τ*_0_ ≈ 4.3 × 10^−15^ and α = 0.3 with *E* in the unit of meV. It can be seen that, at this doping concentration, the Fermi energy is below the threshold energy that the intervalley scattering happens; therefore, the intervalley scattering has no significant effect on the carrier transport.

It is known that the density of states (DOS) scales with energy too. For example, in case of one, two, and three dimensional material systems the energy dependency of DOS is as *E*^−1/2^, *E*^0^, and *E*^1/2^, respectively. For further simplicity, we assumed that *Eg*(*E*)*τ*(*E*) is scaled with energy as *Eg*(*E*)*τ*(*E*) = *g*_0_*τ*_0_*E*^*α_t_*^. As we will discuss, depending on the sign of the scattering exponent α_t_, the multivalley band structure can have detrimental or beneficial effects on the thermoelectric properties of materials.

**Figure 3:**
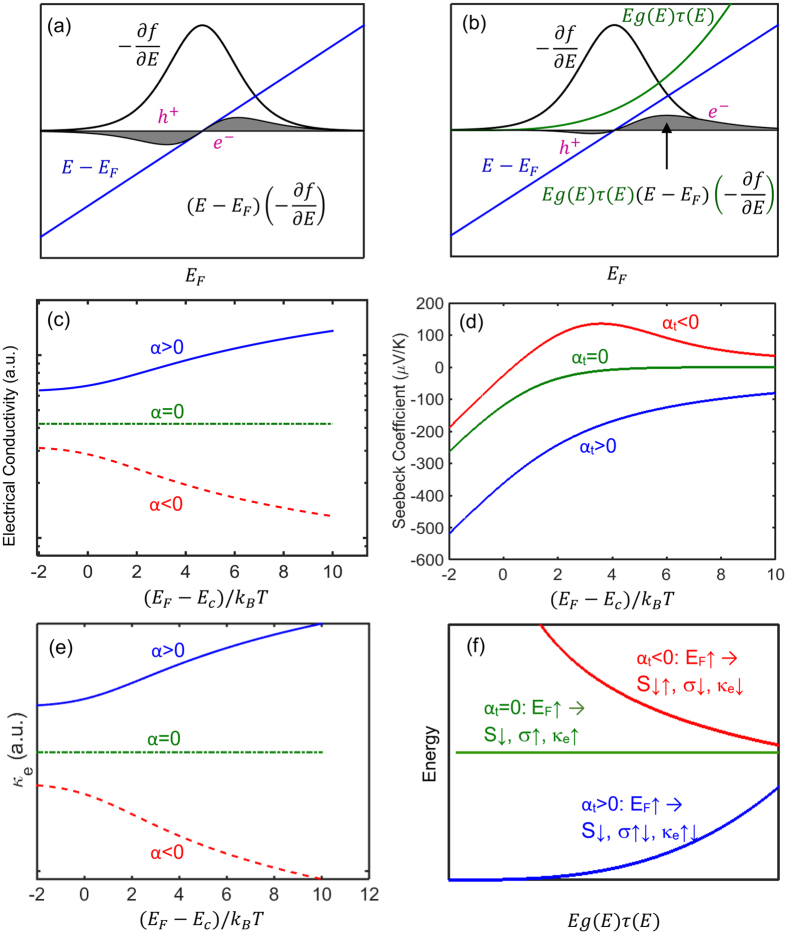
The integral in the numerator of the equation of the Seebeck coefficient (eq.3) consists of (E − E_F_)σ_s_, where σ_s_ ≡ Eg(E)τ(E)(−df/dE) is the spectral conductivity. (**a**) (E − E_F_)(−df/dE) versus E, which is an odd function around E_F_ and does not contribute in the Seebeck coefficient. (**b**) Eg(E)τ(E) versus E, which is an asymmetric function around E_F_ and its existence results in a non-zero Seebeck coefficient. (**c**) Seebeck coefficient versus Fermi energy for three different values of the total energy exponent α_t_. (**d**,**e**) The electrical conductivity and electronic thermal conductvity versus Fermi energy for different signs of the relaxation time exponent α. (**f**) The energy dependency of Eg(E) *τ* (E) for various values of total scattering exponents α_t_ shows how the different quantities change with variation of the Fermi energy. The dependency of quantities to the change of Fermi energy and ***Eg***(*E*)*τ*(*E*) are represented by downward and upward arrows in panel (**f**).

Equation set 3 indicates that the Seebeck coefficient depends on the absolute slope of *Eg(E)τ(E)* around the Fermi energy, i.e., the larger the variation of the *Eg(E)τ(E)* around E_F_, the larger the Seebeck coefficient. The comparison of Fig. 3(a,b) depicts schematically this matter. However, according to eq. 4, the carrier mobility depends on the ratio of the two integrals. The numenrator is 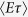 and the denumerator is 〈*E*〉. In order to separate the effect of carrier concentration, in the present discussions, we assume that the carrier concentration is constant and consider the changes due to only the band degeneracy and intervalley scattering. Therefore, the electrical conductivity follows similar trends as the carrier mobility according to eq. 6. The Lorentz number (eq. 5) is not a strong function of the band degeneracy and relaxation time; therefore, it also follows similar trends as the electrical conductivity, hence the carrier mobility, according to eq. 6.

Following the aforementioned equations, several different behaviors of Seebeck coefficient, electrical conductivity, and the electronic thermal conductivity are distinguishable as schematically shown in Fig. 3(c–f).

Figure 3(c) shows the dependency of the electrical conductivity on the relaxation time energy exponent α. The plot assumes that the carrier concentration is constant. In this case, the electrical conductivity increases with Fermi energy if α > 0, remains constant if α = 0, and decreases with Fermi energy if α < 0.

Figure 3(d) illustrates how the trend of Seebeck coefficient versus Fermi energy depends on the value of the exponent α_t_, i.e. the energy scaling exponent of *Eg*(*E*)*τ*(*E*). If α_t_ ≥ 0, the absolute value of the Seebeck coefficient decreases with Fermi energy and saturates. The Seebeck coefficient has more complicated trend when α_t_ < 0. In this case, first, at non-degenerate doping levels (E_F_ − E_c_ < 0) the absolute Seebeck coefficient decreases down to zero and changes sign as the Fermi level enters the conduction band. Then S grows and reaches a peak when E_F_ is a few k_B_T inside the band. As the Fermi energy increases (degenerate doping levels), the Seebeck coefficient drops after the peak and approaches zero again.

Figure 3(c,d) interestingly show that in some ranges of energy, the electrical conductivity and absolute Seebeck coefficient can have similar trend with the change of the Fermi energy. Such a trend contradicts with the typical behavior that the absolute Seebeck coefficient and the electrical conductivity follow opposite trends with the change of the Fermi energy. For example, with the increase of the carrier concentration, Fermi energy and electrical conductivity increase, but the absolute Seebeck coefficient decreases. It should be reminded that, in the present study, the carrier concentration is fixed and the change of the Fermi energy is due to the multivalley effect. For −3/2 < α < 0, we have α_t_ > 0 (as α_t_ = α + 3/2); therefore, both the electrical conductivity and the absolute Seebeck coefficient increase with the reduction of the Fermi energy. As we will discuss later, this case happens strongly for the thermoelectric material Mg_2_Si. When α < −3/2, α_t_ < 0 and the Seebeck coefficient changes sign as E_F_ increases and enters the conduction band. In this range of Fermi energy, again electrical conductivity and absolute Seebeck coefficient have similar trends with both decreasing by further increase of the Fermi energy. However, such a similar behavior, due to its negative slope, is not beneficial for enhancing the thermoelectric power factor.

The trends of changes for both the electrical conductivity (σ) and electronic thermal conductivity (κ_e_) versus Fermi energy depend on the relexation time energy exponent α (not α_t_), and they have a less complicated behavior compared to the Seebeck coefficient. When α > 0, with the increase of E_F_, σ and κ_e_ both increase; when α = 0, σ and κ_e_ remain constant with respect to E_F_; when α_t_ < 0, with the increase of E_F,_ σ and κ_e_ both decrease.

Figure 3(e) depicts similar behavior for the electronic thermal conductivity as that of the electrical conductivity, which, as discussed, is due to weak dependency of the Lorentz number to the Fermi energy. Figure 3(f) summarizes different trends of the electrical conductivity and the Seebeck coefficient versus the Fermi energy and the total energy scaling exponent of *Eg(E)τ(E)*, i.e. α_t_.

Although the degeneracy of the band structure enhances the Seebeck coefficient, it also introduces two deteriorating effects on the carrier mobility: (1) all carrier scattering mechanisms increase by the degeneracy of the band due to the more available number of states for the carries to scatter into, and (2) a new scattering mechanism is enabled, i.e. the intervalley scattering among the degenerate states. Both these effects would reduce the carrier mobility; hence, the electrical conductivity. The reduction of the carrier mobility may be comparable or even more than the effect of the Seebeck coefficient; therefore, the thermoelectric power factor may decrease or increase due to the degenerate bandstructure.

Another indirect effect of the multivalley band structure appears in the total thermal conductivity where both the electronic and lattice parts of thermal conductivity are influenced. The electronic part of thermal conductivity depends on the electrical conductivity and will follow the same trend as that of the electrical conductivity.

It is also important to consider the effect of multivalley band structure on the lattice part of the thermal conductivity, which is often overlooked. The lattice contribution of the thermal conductivity is affected through the phonon-electron scattering mechanism. In general, with the increase of the degeneracy of the band, the scattering of phonons by electrons increases due to the increased electronic density of states^38^. In particular, *N*_*v*_ can affect the lattice thermal conductivity through phonon-electron scattering. Phonon-electron scattering rate increases with *N*_*v*_ and the Fermi energy *E*_*F*_. Moreover, *E*_*F*_ is also a function of *N*_*v*_ and reduces with *N*_*v*_. With the increase of *N*_*v*_, the phonon-electron scattering increases proportionally, but *E*_*F*_ is reduced slightly as *E*_*F*_ is a slow-varying function of *N*_*v*_. Therefore, the phonon-electron scattering rate increases with *N*_*v*_ resulting in smaller lattice thermal conductivity. This scattering mechanism, although small in non-degenerate semiconductors, affects the thermal conductivity of most thermoelectric materials due to their high doping concentration.

**Figure 4:**
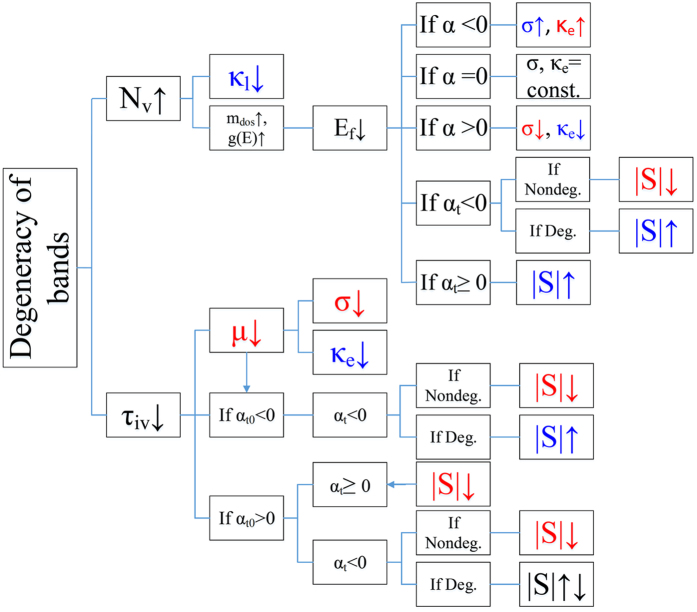
The flowchart of the valleytronics parameters that classifies the trends through which the transport properties are influenced. The increment of the degeneracy of bands means that the band multiplicity increases while the intervalley scattering raises and each effect, causes its own chain of cause and effect. The beneficial and detrimental effects are shown with blue and red colors, respectively. The direction of the arrows show if the quantity is increased or decreased. |S| in the diagram is the absolute value. Deg. and Nondeg. refer to the degenerate and non-degenerate doping levels, respectively.

The classification of the different effects of the valleytronics parameters in thermoelectricity is summarized in the flowchart of Fig. 4. The flowchart shows the chain of cause and effects through which the transport properties are affected. α_t0_ is a new parameter in the flowchart, which is the total energy scaling exponent of *Eg(E)τ(E)* when *τ(E)* excludes the intervalley scattering time, i.e. the total scattering time prior to the inclusion of the intervalley scattering. Since the energy exponent of the intervalley relaxation time is negative, if α_t0_ < 0, α_t_ < 0. If α_t0_ > 0, α_t_ can be negative or positive depending on the strength of the intervalley scattering rate. In many good thermoelectric materials, acoustic phonon scattering is dominant above room temperature; thertefore, α ~ −0.5 and α_t_ > 0. The application of the flowchart is exemplified for several different multivalley semiconductors in the following discussion.

## Results and Discussion

To exemplify the classification method introduced above, we calculated the thermoelectric properties of several different types of materials. The selected materials are Al_x_GaAs_1−x_, Mg_2_Si, Si_0.8_Ge_0.2_, and type-VIII clathrate Si_46_. We employed a multi-band Boltzmann transport theory to calculate their electrical and thermal properties and analyze the advantages and disadvantages of the multivalley band structure and the effects of the intervalley scattering.

In order to determine both the charge carrier and the phonon transport characteristics of the selected materials, the calculations were based on a unique set of energy band structure parameters, lattice properties, carrier concentration, composition, and temperature adopted from refs 13,14,41,42. The adopted model accounts for one valance band (Γ) and three conduction bands (Γ, L, X) for Al_x_Ga_1−x_As, two valence bands (light and heavy holes in Γ) and three conduction bands (2 X and L) for Mg_2_Si, two valence bands (light and heavy holes in Γ) and two conduction bands (X and L) for Si_0.8_Ge_0.2_, and five valance (N, P, NH, ΓH, Γ) and three conduction band minima (ΓH, NH, Γ) for Si_46_-VIII. To demonstrate the effect of intervalley scattering, the transport properties were calculated with and without the inclusion of the intervalley relaxation time. All the bands near the Fermi energy (within 20 k_B_T) were included in the transport properties to incorporate the effect of multiband structure. The calculated properties were derived for optimum value of carrier concentration for each material system. In the subsequent sections, we will present the results of our calculations for the selected materials.

### Al_x_Ga_1−x_As

**Figure 5:**
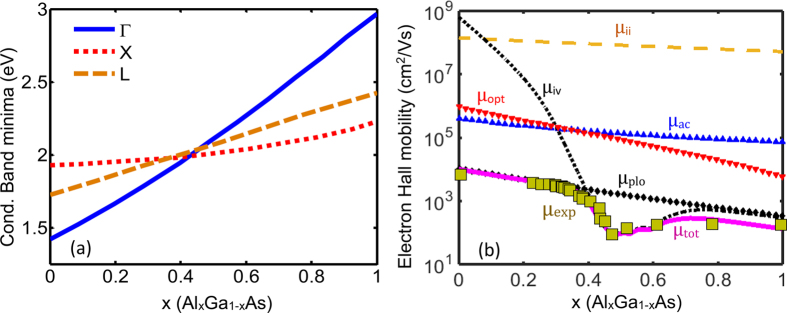
(**a**) Conduction band minima at Γ, L, and X points versus Al content x for Al_x_Ga_1−x_As material system. (**b**) The calculated Hall mobility (solid line) versus x and comparison with empirical data (symbols).

The variation of the conduction band minima at Γ, L, and X points versus Al content, x is demonstrated in Fig. 5(a). The three conduction bands meet each other near x = 0.42. Figure 5(b) which was taken from ref. 14 illustrates the fitted Hall mobility curves to the empirical one (olive squares). The components of the calculated Hall mobility curve have been shown as well. The components represent the contribution of different scattering mechanisms such as the acoustic, ionized impurity, polar longitudinal optical phonons, intervalley scattering, and deformation potential of optical phonons into the electron mobility. The good agreement of the calculated values with the experimental data over the entire range of the composition evidences the model reliability^14^.

**Figure 6:**
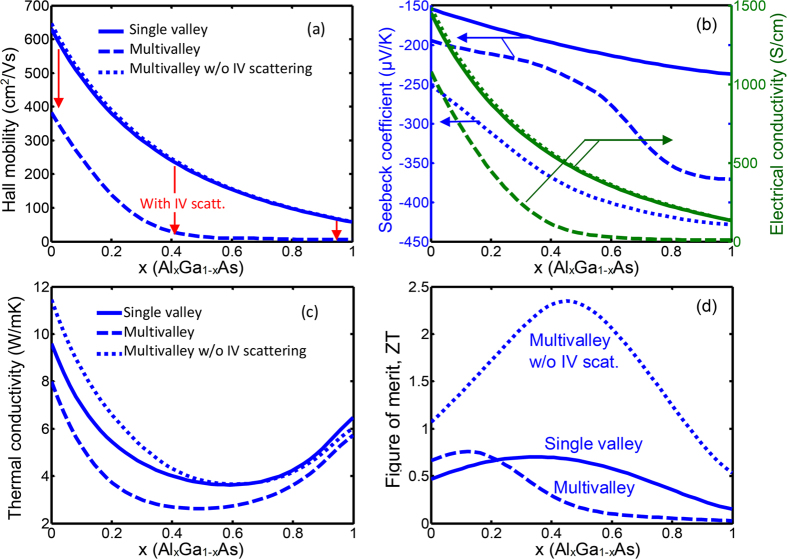
The Hall mobility, electrical conductivity and Seebeck coefficient, thermal conductivity, and figure-of-merit ZT are presented versus Al content of Al_x_Ga_1−x_As material system. The solid, dashed, and dotted lines refer to single valley, multivalley, and multivalley without the intervalley (IV) scattering contribution, respectively.

Figure 6(a–d) illustrate the Hall mobility, Seebeck coefficient, electrical conductivity, thermal conductivity, and the figure-of-merit ZT of AlGaAs compound versus Al content x. Three distinct behaviors corresponding to the contribution of single valley, multivalley, and multivalley without intervalley scattering are shown in Fig. 6(d). Here single valley refers to the lowest conduction band edge, which is the Γ point with degerenreacy of one. In the figures, the blue solid, dashed and dotted curves are indicative of the single valley, multivalley and multivalley without intervalley contributions to the selected properties, respectively. It should be noted that the calculations were performed for a fixed doping concentration (1.44 × 10^19^ cm^−3^) and temperature (1300 K). The Fermi energy is a function of x and decreases from 2.2 k_B_T above the conduction band edge at x = 0 to 0.22 k_B_T at x = 1. A change in the degeneracy of the band affects the density of states and the location of the Fermi energy. The different trends observed in these plots can be explained as follows.

### Hall mobility

The Hall mobility of the single valley is much higher than that of the multivalley indicating the dominancy of the intervalley scattering in electron transport in AlGaAs. However, it can be seen that the Hall mobility of the multi-valley without intervalley scattering is almost same as that of the single valley, which indicates that the degeneracy of the band (*N*_*v*_) by itself, i.e. without intervalley scattering, does not significantly affect the carrier mobility. It is also seen that the mobility reduction due to intervalley scattering is most significant near x = 0.4. This can be attributed to the convergence of the Γ, L, and X bands near this composition that enhances the intervalley scattering.

### Electrical conductivity

The electrical conductivity is a function of charge carrier concentration and carrier mobility. Through the carrier mobility, it also depends on the degeneracy and relaxation time according to *σ* = *eN*_*d*_*μ* (*N*_*v*_, *τ*). Moreover, the carrier concentration *N*_*d*_ is a function of both the Fermi energy *E*_*F*_ and the degeneracy *N*_*v*_, i.e. *N*_*d*_ = *N*_*d*_ (*E*_*F*_, *N*_*v*_). Therefore, when the carrier concentration is fixed, the electrical conductivity follows the trend of the carrier mobility. It can be seen from Fig. 6(b) that the electrical conductivity follows the same trend as of the Hall mobility in all cases.

### Seebeck coefficient

In Fig. 6(b), arrows indicate the vertical axis to which the curves are corresponded. Figure 6(b) illustrates that even though the intervalley scattering reduces the absolute value of the Seebeck coefficient of AlGaAs, the effect of multivalley contribution through *N*_*v*_ is still beneficial over the entire range of the alloy composition. The absolute Seebeck coefficient increases up to 50% at x = 1. Interestingly, for 0.6 < x < 0.8, as the Al fraction increases, the absolute Seebeck coefficient is enhanced more rapidly and it almost saturates when x > 0.8. In addition, it can be seen that once the effect of the multivalley scattering is removed, the absolute Seebeck coefficient increases significantly and keeps increasing with the Al fraction.

### Thermal conductivity

It is interesting to note that even though the Hall mobility values for the cases of the single valley and multi-valley without intervalley scattering are very similar, the thermal conductivity of the two cases are different for small Al contents (x < 0.4). This may be strange at first glance, but can be understood by considering the effect of phonon electron scattering. This observed difference is mainly due to the effect of the degeneracy of the band *N*_*v*_, which increases the phonon-carrier scattering and reduces the lattice part of the thermal conductivity. The phonon-electron scattering is often important at low temperature. With the temperature increase, the three-phonon scattering increases and usually dominates the phonon-electron scattering.

The thermal conductivity of the multivalley and multivalley without intervalley scattering are also different due to the difference in the electronic part of the thermal conductivity, which follows the difference of the Hall mobilities. Due to the large electrical conductivity of AlGaAs material system, compared with many good thermoelectric materials, the electronic contribution of the thermal conductivity is important.

### Figure of merit

Although the peak ZT does not show significant difference in cases of the single valley and multivalley, the peak ZT happens at distinct places on the x-axis. The peak ZT for the multivalley plot occurs at a smaller x value (x ~ 12–16%) compared to that of the single valley (x ~ 0.4). For the case of multivalley without the intervalley scattering, the ZT peak is much higher than that of previous two peaks. The peak is located almost at the same place as that of the single valley, which is approximately where the bands converge. This indicates that the intervalley scattering has significantly reduced the ZT of AlGaAs. In summary, in AlGaAs material system, the multivalley contribution has both detrimental and beneficial effects. The beneficial effect is the reduction of the thermal conductivity through the reduction of both the lattice part and the electronic part. The detrimental effect is the reduction of the thermoelectric power factor by significantly reducing the electrical conductivity. Overall, the multivalley effect on ZT is positive for x < 0.2 although very small. At x > 0.2, the effect of the multivalley bandstructure is negative and reduces the ZT. The detrimental effect of the multivalley contribution is the results of the intervalley scattering which not only decreases the carrier mobility, but also lowers the Seebeck coefficient due to the negative sign of the scattering exponent. The later one has more significant effect on decreasing the power factor contrary to the general belief.

### Mg_2_Si

**Figure 7:**
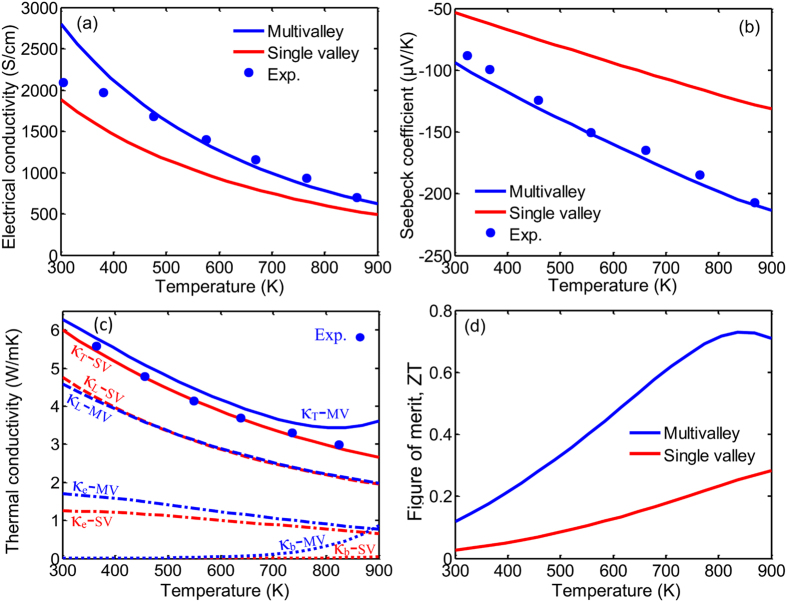
The main thermoelectric properties of Mg_2_Si compound versus temperature at electron concentration of 6.5 × 10^19^ cm^−3^. The red and blue curves correspond to the hypothetical single valley and the multivalley cases, respectively. The solid, dashes, dash-dotted, and dotted curves in the thermal conductivity plot correspond to the total κ_T_, lattice part κ_L_, electronic part κ_e_, and bipolar part κ_b_, respectively.

The compounds of Mg_2_X (where X = Si, Ge, and Sn) and their solid solutions have been of interest recently due to their high thermoelectric preformance. Mg_2_Si has a degenerate conduction band minima with degeneracy of three at X point. The thermoelectric porperties of Mg_2_Si and its nanostructured form has been recently modeled versus doping and temperature variations^41^,^42^,^43^,^44^. The variation of the main thermoelectric properties of Mg_2_Si compound versus temperature at electron concentration of 6.5 × 10^19^ cm^−3^ is presented in Fig. 7. The Fermi energy is a function of temperature and reduces from 3.3 k_B_T above the conduction band edge at room temperature to 0.14 k_B_T below the conduction band edge at 900 K. The experimental data taken from ref. [45] is also shown with symbols. The multivalley solution is in good agreement with the experimental results. Figure 7(a) shows that the electrical conductivity of both the single valley and the multivalley Mg_2_Si decreases as the temperature increases; however, the electrical conductivity of the single valley remains always lower than that of the multivalley. Here single valley refers to the lowest conduction band edge, which is in the X point, with hypothetically setting its degerenreacy equal to one, i.e. choosing *N*_*v*_ = 1.

Figure 7(a) shows the comparison of the electrical conductivity for the two cases of the multivalley and single valley conduction bands. In Mg_2_Si, due to the dominancy of the acoustic phonon scattering, the effect of intervalley scattering on transport properties is negligible. Therefore, the decrease in the electrical conductivity of Mg_2_Si alloy versus temperature originates dominantly from the acoustic phonon scattering. The single valley curve of the electrical conductivity lies below the multivalley curve of electrical conductivity, which is mainly due to the higher carrier mobility of the multivalley bandstrcuture case. The dominant carrier scattering in Mg_2_Si is by acoustic phonons, which means α = −1/2 or α_t_ = 1. For a fixed carrier concentration, the multivalley bandstrcuture has lower Fermi energy (due to higher density of states effective mass). Therefore, according to flowchart displayed in Fig. 4, the electrical conductivity increases in multivalley bandstructure. Interestingly, as shown in Fig. 7(b), the absolute Seebeck coefficient also increases in the multivalley bandstrcuture. The reduction of the Fermi energy enhances the absolute Seebeck coefficient significantly over the whole range of the temperature. The largest discrepancy between the two curves occurs near 900 K.

Figure 7(c) demonstrates that the band degeneracy in Mg_2_Si compound results in slightly higher thermal conductivity, which is due to the higher electronic part compared to single valley case. Also, the bipolar part of thermal conductivity increases due to the reduction of the Fermi energy, which explains the increasing slope of the thermal conducvtity at high temperature (>800 K).

Figure 7(d) shows the overall effect of the band degeneracy on ZT of Mg_2_Si versus temperature. The dimensionless figure-of-merit ZT shows a peak at approximately 850 K for multivalley case while the single valley case has no peak and increases as temperature raises. However, the ZT of multivalley case is over 3-fold larger than that of the single valley one at that temperature.

### Si_0.8_Ge_0.2_

**Figure 8:**
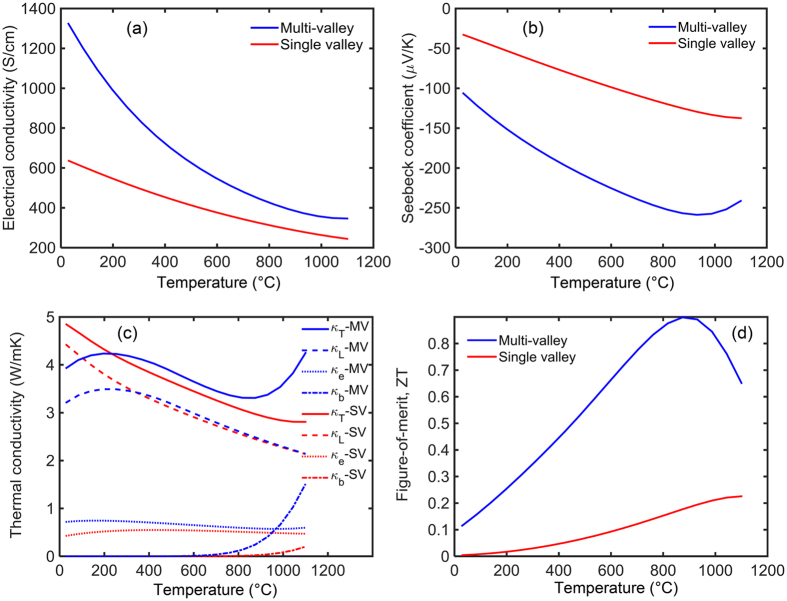
The main thermoelectric properties of SiGe compound versus temperature. The red and blue curves correspond to the single valley and the multivalley cases, respectively. The dashes and dotted curves in the thermal conductivity plot correspond to the lattice part and electronic part, respectively.

The variation of the main thermoelectric properties of SiGe compound versus temperature is presented in Fig. 8. Here, the calculations were achieved for fixed carrier concentration of 10^20^ cm^−3^. The Fermi energy varied from 2.2 k_B_T above the conduction band edge at room temperature to 1.2 k_B_T below the conduction band edge at 1100 °C. Figure 8(a) shows that the electrical conductivity of both the single valley and the multivalley SiGe materials decreases as the temperature increases in most of the temperature range (200–1100 K). Here single valley refers to the lowest conduction band edge, which is near the X point, with hypothetically setting its degerenreacy equal to one, i.e. choosing *N*_*v*_ = 1. In SiGe, carrier scattering by acousatic phonons is also dominant and the intervalley scattering is negligible. Therefrore, the effect of *N*_*v*_ on electrical conductivity is stronger than the effect of τ_iv_. The Fermi energy redcues with the inclusion of the extra valleys (*N*_*v*_ = 6) and, accroding to the flowchart of Fig. 4, the total scattering rate redcues, which in turn increases the electrical conductivity. Above approximately 1100 K, the thermal excitation of carriers leads to the bipolar effect which causes the electrical conductivity to increase with temperature. It is observed that the discrepancy between the electrical conductivity of two cases almost diminishes at 1400 K, which can be associated with the dominancy of the acoustic phonon scattering over the intervalley scattering. Figure 8(b) shows the variation of the Seebeck coefficient versus temperature for the two cases. The degeneracy of the energy band enhances the Seebeck coefficient significantly over the whole range of the temperature. The largest discrepancy between the two curves occurs near 1100 K. Above approximately 1100 K, the absolute Seebeck coefficient reduces following opposite trend as that of the electrical conductivity. The changed slope of the Seebeck coefficient at above 1100 K can be attributed to relatively small band gap of SiGe, comparable to the thermal energy, which allows the bipolar effect to take place at high temperature.

Figure 8(c) shows that the band degeneracy in SiGe compound leads to lower lattice thermal conductivity at low temperature but higher electronic thermal conductivity over the entire range of temperature. As a result, the total thermal conductivity is redcued at lower temperature range (T < 250 °C) but increases at higher temperature. The reduction of the lattice thermal conductivity at lower temperature is due to the enhancement of the phonon scattering by charge carriers as discussed in the flowchart of Fig. 4. As the temperature increases above 1100 K, the thermal conductivity further increases due to the bipolar effect with the thermal conductivity of the multivalley band increasing more rapidly than the single valley. This indicates that the multivalley band enhances the bipolar effect, which can be associated to the larger number of bipolar transport channels in this case.

Figure 8(d) demonstrates the overall effect of the band degeneracy on ZT of SiGe versus temperature. The dimensionless figure-of-merit ZT shows a peak at approximately 1200 K for both the single valley and multivalley cases. However, the ZT of multivalley case is almost 4 times larger than that of the single valley one at that temperature. In SiGe, due to the dominancy of the acoustic phonon scattering, the effect of intervalley scattering on transport properties is negligible. Therefore, the decrease in the electrical conductivity of SiGe alloy originates dominantly from the change of the Fermi level by *N*_*v*_ and not the intervalley scattering.

### Si_46_-VIII

Clathrate Si_46_ type VIII was recently studied for its unusually large number of carrier pockets near both the conduction and valence band edges^46^. It has 19 electron pockets near the conduction band edge and 27 hole pockets near the valence band edge. These numbers are the largest values among all the known good thermoelectric materials. The large density of states near the band edges was further predicted to lead to a giant thermoelectric power factor (>0.004 Wm^−1^K^−2^)^11^. Consequently, the parental Si_46_-VIII was suggested as a good starting material to engineer Si-based clathrate thermoelectric materials and its intercalation with different alkali and alkaline-earth metals was studied and the promising rattlered structures were identified^47^.

**Figure 9:**
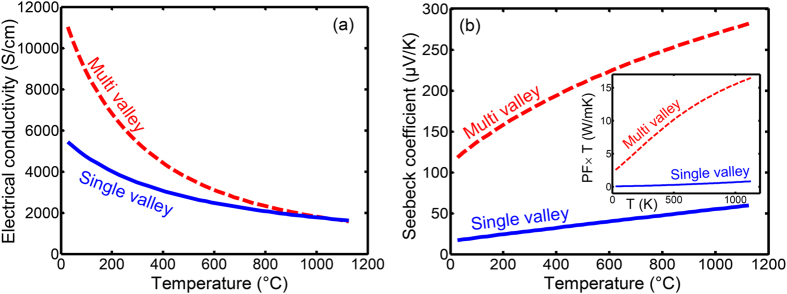
(**a**) The electrical conductivity and (**b**) the Seebeck coefficient of Si_46_-VIII versus temperature. The inset shows the power factor versus temperature.

For comparison with the conventional multivalley materials AlGaAs and SiGe, the transport properties of Si_46_-VIII were also studied versus the effect of the multivalley band structure.. The calculation were performed for fixed carrier concentration of 1.1 × 10^21^ cm^−3^. The Fermi energy altered from 4.9 k_B_T below the valance band edge at room temperature to 0.5 k_B_T below the valance band edge at 1100 °C. Figure 9(a,b) depict the variation of the electrical conductivity and Seebeck coefficient versus temperature for the pristine Si_46_-VIII. Here single valley refers to the lowest conduction band edge, which is a point on the ΓH line, with hypothetically setting its degerenreacy equal to one, i.e. choosing *N*_*v*_ = 1. As before, the blue and red curves refer to single and multivalley cases, respectively. As it can be seen, the multivalley band structure of p-type Si_46_-VIII has a beneficial effect on both electrical conductivity and Seebeck coefficient. For comparison, for AlGaAs, multivalley band structure reduced both the electrical conductivity and the Seebeck coefficient. In SiGe it increased the Seebeck coefficient but decreased the electrical conductivity. The simultaneous enhancement of the electrical conductivity and the Seebeck coefficient contradicts with the general trends that these two quantities behave in opposite ways.

It is also remarkable that as the temperature increases, the beneficial effect of the multivalley in Si_46_-VIII becomes less effective for the electrical conductivity while it becomes more effective for the Seebeck coefficient. Since the intervalley scattering increases with the temperature increment, the carrier mobility decreases; hence, the electrical conductivity decreases. It is normally expected that the increment of the intervalley scattering should also decrease the Seebeck coefficient due to its positive energy exponent. However, due to the existence of a large number of hole pockets near the valence band edge, the temperature increment results in larger contribution of the hole pockets which are not located exactly at the band edge but are at higher energy with a few k_B_T. Therefore, the Seebeck coefficient is affected more positively by the multivalley bandstructure when the temperature increases, which dominates the detrimental effect of the intervalley scattering.

### Comparison of AlGaAs, Mg_2_Si, SiGe, and Si_46_-VIII

**Figure 10:**
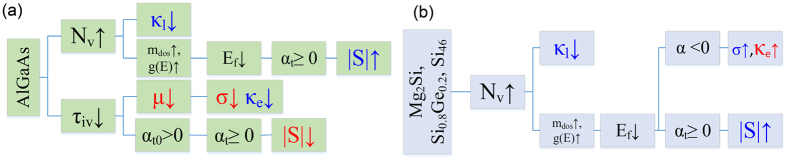
Dominant valleytronics effects are shown for Al_x_Ga_1−x_As (**a**), Mg_2_Si, Si_0.8_Ge_0.2_, and Si_46_-VIII (**b**). The blue letters and arrows indicate the beneficial effects while the red letters and arrows indicate the detrimental effects.

The dominant effects of the degeneracy of the band in AlGaAs, Mg_2_Si, SiGe, and Si_46_-VIII can be summarized as shown in Fig. 10. Here, we have ignored the small effects and have shown only the ones which determine how the transport properties are changed through valleytronics. The left diagram shows how the degeneracy of the band affects the main thermoelectric properties of AlGaAs through either increased *N*_*v*_ or decreased scattering time. The right diagram schematically illustrates the sequence of causes and effects occurred for the main transport properties of Mg_2_Si, Si_0.8_Ge_0.2_, and Si_46_-VIII materials. It can be seen that all three materials follow same sequence of effects. The beneficial and detrimental effects are also color codes by blue colored upward arrow and red colored downward arrows, respectively. When *N*_*v*_ increases, the Fermi energy decreases (assuming carrier concentration is fixed). According to Fig. 3, within a certain range of Fermi energy, which depends on the band properties, the Fermi energy reduction can simultaneously increase both the Seebeck coefficient and the electrical conductivity. Interestingly, this trend is observed for the case of Mg_2_Si, Si_0.8_Ge_0.2_, and Si_46_-VIII. However, in AlGaAs, the electrical conductity is decreased due to the dominacy of the intervalley scattering. The thermal conductivity, including both the lattice and the electronic parts, decreases in AlGaAs. In contrast, in Mg_2_Si and Si_0.8_Ge_0.2_ the total thermal conductivity increases slightly in the temperature range of interest mainly due to the enhancement of the electronic part of the thermal conductivity.

It should be note that, according to well-known Matthiessen’s rule, *τ(E)* is a function of mixed exponents. As discussed, α and α_t_ denote the dominant exponent of *τ(E)* and *Eg(E)τ(E),* respectively, at energies around the Fermi surface. It can be concluded that, in the case of AlGaAs, τ_iv_ has the dominant effect on ZT. However, in the case of Mg_2_Si, Si_0.8_Ge_0.2_, and Si_46_-VIII, the degeneracy of the band *N*_*v*_ is governing the overall effect on ZT.

## Conclusion

The application of valleytronics as means of controlling over the thermoelectric material properties was presented. It was shown that the interplay among the valleytronics parameters such as the degeneracy of the valley *N*_*v*_, intervalley transition time τ_iv_(E), effective mass m_dos_, scattering exponent α_t_, and the Fermi energy E_F_ can result in both beneficial or detrimental effects on the thermoelectric transport properties. The degeneracy of the valley *N*_*v*_ increases the density of states effective mass and reduces the Fermi energy for a given doping concentration. The reduction of the Fermi energy, reduces the average carrier energy which can affect all different scattering mechanisms. Each of these effects may increase or decrease the Seebeck coefficient, charge carrier mobility, and the lattice thermal conductivity. The intervalley scattering reduces both the carrier mobility and the Seebeck coefficient. Therefore, for a given doping concentration, with the increase of the number of valleys, the following conclusions can be drawn:

- (a) The Seebeck coefficient and the electrical conductivity may decrease or increase.
- (b) The average carrier energy always decreases; hence, the acoustic phonon scattering rate decreases and the ionized impurity scattering rate increases. This may increase or decrease the carrier mobility depending on which scattering mechanism is dominant.
- (c) The lattice thermal conductivity may also decrease or increase through the variation of the phonon scattering rate by the charge carriers.
- (d) The ZT may decrease or increase depending on the strength of the different effects.
- (e) For the case of Al_x_Ga_1−x_As, the beneficial effect of the multivalley bandstructure is remarkably reduced due to the strong effect of the intervalley scattering.
- (f) For the case of Si_46_-VIII, due to the large degeneracy of the valleys, the beneficial effect of *N*_*v*_ significantly dominates over the detrimental effects.
- (g) The effect of the intervalley scattering increases as the carrier concentration and the temperature increase.

It was concluded that, although valleytronics can engineer better thermoelectric materials, the bandstructure optimization requires detailed computations to overcome the detrimental effects. The classification of the different trends through which the valleytronics affects the ultimate transport properties was presented in a flowchart that draws the roadmap for designing a thermoelectric material.

## Additional Information

**How to cite this article**: Norouzzadeh, P. and Vashaee, D. Classification of Valleytronics in Thermoelectricity. *Sci. Rep.* **6**, 22724; doi: 10.1038/srep22724 (2016).

## Figures and Tables

**Table 1:** Scattering exponents of various scattering mechanisms and their effect on Seebeck coefficient.

Scattering type	Scattering exponentvalue (α)	Effect on Seebeckcoefficient
Acoustic Phonons	−1/2	S↓
Intervalley	−1/2	S↓
Defect	−1/2	S↓
Carrier−carrier	−1/2	S↓
Piezoelectric	−1/2	S↓
Ionized impurity	3/2	S↑
Polar LO phonons	+ or − depending on E	S↑↓
